# Improvement of Noise Uncertainty and Signal-To-Noise Ratio Wall in Spectrum Sensing Based on Optimal Stochastic Resonance

**DOI:** 10.3390/s19040841

**Published:** 2019-02-18

**Authors:** Di He, Xin Chen, Ling Pei, Lingge Jiang, Wenxian Yu

**Affiliations:** 1 Shanghai Key Laboratory of Navigation and Location-Based Services, Shanghai Jiao Tong University, Shanghai 200240, China; xin.chen@sjtu.edu.cn (X.C.); ling.pei@sjtu.edu.cn (L.P.); 2 Department of Electronic Engineering, Shanghai Jiao Tong University, Shanghai 200240, China; lgjiang@sjtu.edu.cn; 3 Shanghai Key Laboratory of Intelligent Sensing and Recognition, Shanghai Jiao Tong University, Shanghai 200240, China; wxyu@sjtu.edu.cn

**Keywords:** cognitive radio (CR), spectrum sensing, energy detector (ED), signal-to-noise ratio (SNR) wall, optimal stochastic resonance

## Abstract

Noise uncertainty and signal-to-noise ratio (SNR) wall are two very serious problems in spectrum sensing of cognitive radio (CR) networks, which restrict the applications of some conventional spectrum sensing methods especially under low SNR circumstances. In this study, an optimal dynamic stochastic resonance (SR) processing method is introduced to improve the SNR of the receiving signal under certain conditions. By using the proposed method, the SNR wall can be enhanced and the sampling complexity can be reduced, accordingly the noise uncertainty of the received signal can also be decreased. Based on the well-studied overdamped bistable SR system, the theoretical analyses and the computer simulations verify the effectiveness of the proposed approach. It can extend the application scenes of the conventional energy detection especially under some serious wireless conditions especially low SNR circumstances such as deep wireless signal fading, signal shadowing and multipath fading.

## 1. Introduction

In the last decade, owing to the rapid development of wireless communications, such as the rapid deployment of the 3G and 4G mobile communication systems around the world, some old spectrum resource transferring through the market auction in many countries, the amount of information transmitted will exceed the carrying capacity of the existing radio spectrum, the spectrum resource has become very limited, which restricts the progress of high speed wireless communications seriously. Simultaneously, the allocated spectrum has not been utilized effectively, which leads to many spectrum holes in the licensed frequency bands frequently. To enhance the spectrum utility, cognitive radio (CR) networks were proposed to allow the secondary users (SUs) access to the licensed users or primary users’ (PUs) spectrum for transmission [1]. In the CR networks, generally there exists two kinds of spectrum sharing modes, the overlay mode and the underlay mode, while in the overlay mode the CR has some knowledge about existing transmission and may support PU in transmission utilizing the channel for its purposes as well; and in the underlay mode it permits the SUs to transmit even if the channel has been occupied by the PUs already, as long as the interference temperature is under a regulatory limit [2]. In this study, we will focus on the overlay mode.

In the overlay mode, to motivate the SU transmission without interrupting the PU transmission, it will decide the existence of the PU signal in the SU’s receiver end first, say the spectrum sensing, which is a very important task in CR networks. Based on previous studies, it can be found that the spectrum sensing can be realized by non-cooperative sensing methods and cooperative sensing methods [3,4,5,6,7], while the cooperative methods are usually used to overcome the problem of shadow fading in wireless communications. Generally, the non-cooperative sensing methods are still the basis of the cooperative sensing methods. Within various kinds of non-cooperative sensing methods, the energy detection [4], feature detection [5], and matched-filter detection [6] are the most studied methods, in which energy detection attracts a lot of research interest because it is easy to use and no a prior knowledge of PU signal is required in the sensing process. However, at the same time, the signal-to-noise ratio (SNR) wall problem [8] seriously restricts the application of the traditional energy detection, which implies that the number of samples at the receiver may increase rapidly to reach a certain high threshold to fulfill a higher detection probability under a constant false alarm rate (CFAR), if there exists noise uncertainty in the SU’s receiver in the sensing process. The SNR wall problem degrades the performance of energy detection especially under low SNR, which always appears in wireless communications.

Besides the above studies and research, the survey in [9] briefly described the ways for obtaining energy-efficiency in cooperative sensing, and it also summarized the algorithms leading to relative energy saving while assuring high sensing performance in terms of the global probability of detection or the global probability of false alarm. In [10], several topics and open problems worth mentioning in spectrum sensing for CR were also proposed, such as quick detection, adaptive sensing and learning, joint spectrum sensing and efficient resource utilization. While for the SNR wall problem, the detection performance of generalized energy detector was studied in [11] under different distributions of noise uncertainty and the problem of opportunistic spectrum access using full-duplex radios in the presence of unknown PU channel statistics was studied in [12]. In [13] the recent advances in the spectrum sensing framework as the main enabling technology for the interweaving cognitive radio model is provided. References [14,15] derived the closed form expressions for the critical frame length that equalizes the energy consumption and energy efficiency in CR schemes, which has been validated by the simulation results.

To overcome the SNR wall problem stated above, a novel non-cooperative spectrum sensing approach based on the combination of the dynamic analysis method of stochastic resonance (SR) and the traditional energy detection is proposed in this study. It utilizes the special property of the SR system that can improve the SNR of the SR system especially under certain conditions [16,17,18,19]. By introducing an optimal SR system as the pre-processor of the traditional energy detector, the SNR wall can be improved and the number of samples at the receiver can be reduced effectively, which results in the improvement of detection performance in CR networks. The optimal driving parameters of the SR system can also be found through the optimization process. Theoretical analyses verify the effectiveness of the proposed approach. The computer simulations also show that the SNR wall problem can be solved effectively especially under low SNR circumstances.

The remaining part of this paper is arranged as follows: Section 2 explains the traditional non-cooperative energy detection method and corresponding SNR wall problem briefly. In Section 3, the SR-based energy detection approach is proposed and discussed together with its optimization process. The theoretical performance improvement analyses are given in Section 4 in details based on a well-known overdamped bistable SR system. Computer simulation results are given in Section 5 to verify the effectiveness of the proposed approach, and the comparison results with the traditional spectrum sensing method are also presented. Finally the concluding remarks are summarized in Section 6.

## 2. Traditional Detection Methods and the Signal-To-Noise ratio Wall Problem

Within many different non-cooperative spectrum sensing methods, such as cyclostationary detection, the covariance-based detection, the matched-filter detection etc. [4,5,6], energy detection has the lowest computational cost and is the easiest to be used. Simultaneously, most detection methods should know a prior knowledge of the PU signal except the energy detection, which also guarantees that energy detection is a good property that can be applied in real applications.

Generally speaking, a spectrum sensing task can be abstracted as the following two-hypotheses testing problem

(1) $$\{\begin{array}{cc} H_{0}: & r(k)=n(k),\\ H_{1}: & r(k)=h(k)\cdot s(k)+n(k),\;(k=1,2,\cdots ) \end{array}$$

where *r*(*k*) is the received real signal at the SU’s receiver, *s*(*k*) is the PU signal, *n*(*k*) is the additive channel noise, and *h*(*k*) is the time-varying fading factor under wireless transmission circumstances. To simplify the analyses in this this study, it can always be assumed that *s*(*k*) obeys a certain distribution with mean 0 and variance $\sigma_{s}^{2}$; *n*(*k*) obeys Gaussian distribution with mean 0 and variance $\sigma_{n}^{2}$, which is independent of *s*(*k*) and can be regarded as a sum of additive channel noise, thermal noise, co-channel multi-user interference and so on. *h*(*k*) obeys Rayleigh distribution with mean $\bar{h}$ and variance $\sigma_{h}^{2}$, which is independent of *s*(*k*) and *n*(*k*). Because the time period of spectrum sensing frame is relatively shorter than the time period of transmission, it can be assumed that $h(k)\equiv \bar{h}$ is a constant in the theoretical analyses and computer simulations thereafter.

In the traditional detection method, the SU’s receiver calculates the power *A*(*r*) of the received signal *r*(*k*) as follows

(2) $$A\left(r\right)=\frac{1}{K}\sum_{k=1}^{K}r^{2}\left(k\right)$$

where *K* is the total number of samples at the SU’s receiver. Then by comparing the statistic *A*(*r*) with a fixed threshold value *γ_ED_*, one can decide whether the PU signal exists or not, which can be expressed as

(3) $$A(r)\begin{array}{c} H_{1}\\ >\\ <\\ H_{0} \end{array}\gamma_{ED}$$

The threshold *γ_ED_* is often chosen to guarantee the CFAR property of the traditional detector. So the detection probability *P_d_*_(*ED*)_ and the false alarm rate *P_fa_*_(*ED*)_ of the traditional detection method can be calculated by the following two equations, respectively

(4) $$P_{d(ED)}=\mathrm{Pr}\left\{A(r)>\gamma_{ED}|H_{1}\right\}$$

(5) $$P_{fa(ED)}=\mathrm{Pr}\left\{A(r)>\gamma_{ED}|H_{0}\right\}$$

where Pr{⋅} is the probability of the event.

From (2), it can be derived that

(6) $$\{\begin{array}{cc} H_{0}: & \begin{array}{c} \begin{array}{l} E\left[A\left(r\right)\right]=\sigma_{n}^{2},\\ \mathrm{var}\left[A\left(r\right)\right]=\frac{2}{K}\sigma_{n}^{4}; \end{array} \end{array}\\ H_{1}: & \begin{array}{c} \begin{array}{l} E\left[A\left(r\right)\right]={\bar{h}}^{2}\cdot \sigma_{s}^{2}+\sigma_{n}^{2},\\ \mathrm{var}\left[A\left(r\right)\right]=\frac{2}{K}\cdot {\left({\bar{h}}^{2}\cdot \sigma_{s}^{2}+\sigma_{n}^{2}\right)}^{2} \end{array} \end{array} \end{array}$$

where E[⋅] and var[⋅] are the mean and variance functions, respectively. Therefore, (4) and (5) can also be rewritten as

(7) $$P_{d(ED)}=Q_{\chi_{K}^{2}}\left(\frac{\gamma_{ED}}{\sigma_{n}^{2}}\right),$$

(8) $$P_{fa(ED)}=Q_{\chi_{K}^{2}}\left(\frac{\gamma_{ED}}{{\bar{h}}^{2}\cdot \sigma_{s}^{2}+\sigma_{n}^{2}}\right),$$

where $Q_{\chi_{K}^{2}}\left(\cdot \right)$ is the right-tail probability of the central chi-squared probability density function (pdf) under K degrees of freedom. To guarantee the detection performance under a certain CFAR, the threshold *γ_ED_* can be determined by

(9) $$\gamma_{ED}=\left({\bar{h}}^{2}\cdot \sigma_{s}^{2}+\sigma_{n}^{2}\right)\cdot Q_{\chi_{K}^{2}}^{-1}\left(P_{fa(ED)}\right),$$

where $Q_{{}_{\chi_{K}^{2}}}^{-1}\left(\cdot \right)$ is the inverse function of $Q_{\chi_{K}^{2}}\left(\cdot \right)$. In real applications, the parameters ${\bar{h}}^{2}$, $\sigma_{s}^{2}$ and $\sigma_{n}^{2}$ in (9) can be replaced by corresponding estimates ${\hat{\bar{h}}}^{2}$, ${\hat{\sigma }}_{s}^{2}$ and ${\hat{\sigma }}_{n}^{2}$, respectively. In the following analyses, for simplicity, we assume that both ${\bar{h}}^{2}$ and $\sigma_{s}^{2}$ are known or can be estimated unbiasedly at the SU’s receiver, so the performance is only determined by the noise variance estimate ${\hat{\sigma }}_{n}^{2}$.

According to the Central Limit Theorem [3], when the total number of samples *K* at the SU’s receiver is big enough, (7) and (8) can be approximated by

(10) $$P_{d(ED)}\approx Q\left(\frac{A\left(r\right)-{\hat{\sigma }}_{n}^{2}}{{\hat{\sigma }}_{n}^{2}}\right),$$

(11) $$P_{fa(ED)}\approx Q\left(\frac{A\left(r\right)-{\bar{h}}^{2}\cdot \sigma_{s}^{2}-{\hat{\sigma }}_{n}^{2}}{{\bar{h}}^{2}\cdot \sigma_{s}^{2}+{\hat{\sigma }}_{n}^{2}}\right),$$

where Q(⋅) is the standard Gaussian complementary cumulative distribution function (CDF) whose definition can be found in [16].

Due to the uncertainty of the wireless channel in CR networks, there may exist some difference between ${\hat{\sigma }}_{n}^{2}$ and $\sigma_{n}^{2}$, or there may be a distributional uncertainty of the noise power within the following interval [8]

(12) $${\hat{\sigma }}_{n}^{2}\in \left[\frac{1}{\rho }\sigma_{n}^{2},\rho \sigma_{n}^{2}\right],$$

where ρ>1 is a positive parameter which defines the size of the noise uncertainty [8]. Then the sampling complexity of the conventional detection method *N_ED_* is [8].

(13) $$N_{ED}=\frac{2{\left[Q^{-1}\left(P_{fa(ED)}\right)-Q^{-1}\left(1-P_{d(ED)}\right)\right]}^{2}}{{\left[SNR_{i}-\left(\rho -\frac{1}{\rho }\right)\right]}^{2}},$$

where *SNR_i_* is the SNR of *r*(*k*) under *H*_1_ in (1), which can be theoretically calculated by

(14) $$SNR_{i}=\frac{{\bar{h}}^{2}\sigma_{s}^{2}}{\sigma_{n}^{2}}.$$

From (13) it can be found that when *SNR_i_* is reducing and approaching $\left(\rho -\frac{1}{\rho }\right)$ gradually, the sampling complexity *N_ED_* will approach infinity, which implies that the spectrum sensing performance of *P_d_*_(*ED*)_ under certain *P_fa_*_(*ED*)_ cannot be guaranteed even if the number of samples is big enough. So it is also called an SNR wall as

(15) $$SNR_{wall}^{\left(ED\right)}=\rho -\frac{1}{\rho }.$$

In other words, when *SNR_i_* is lower than the SNR wall defined above, it is impossible for the conventional detector to reach the certain spectrum sensing performance. However, the low SNR condition is a very common circumstance in wireless CR networks, so it is a very serious problem which restricts the application of the conventional detection method.

## 3. Optimal Stochastic-Resonance-Based Detection Approach under Low Signal-To-Noise Ratio

To overcome the SNR wall problem mentioned in the last Section, a novel spectrum sensing approach based on the optimal SR technique and the conventional detection method was proposed, especially for the application under low SNR.

**Figure 1:**
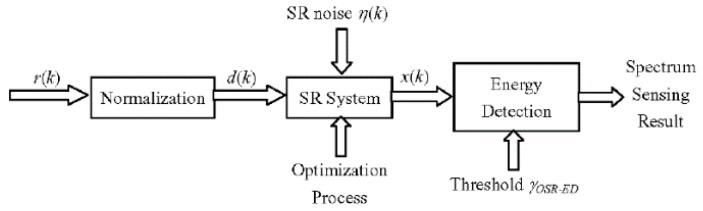
Block diagram of the proposed optimal stochastic resonance-based detector.

Firstly, to give an intuitive explanation, we give the block diagram of the proposed optimal SR-based detector in Figure 1. The received signal *r*(*k*) is first normalized to fulfill the input signal requirement of the SR system, that is

(16) $$d\left(k\right)=\frac{1}{\sqrt{\frac{1}{K}\sum_{k=1}^{K}r^{2}\left(k\right)}}\cdot r\left(k\right),\;(k=1,2,\cdots )$$

where *d*(*k*) is the normalization of the signal *r*(*k*).

Use *d*(*k*) as one of the driving inputs to the SR system, and introduces a pseudo random noise *η*(*k*) with mean 0 and variance $\sigma_{\eta }^{2}$ as another driving input to the SR system, which is independent of *d*(*k*) as shown in Figure 1, then the dynamic equation of the SR system can be expressed by

(17) x(k+1)=f[x(k),d(k),η(k),P(k)],

where f[⋅] is the nonlinear dynamic function of the SR system; *x*(*k*) is the state variable of the SR system; and ***P***(*k*) is the parameter vector of the selected SR system f[⋅].

As is known, SR is a kind of nontrivial behavior in nonlinear systems with the influence of noise. It reveals the phenomena that the ordered response of a dynamic system with weak input signals can be significantly increased by appropriately tuning the noise intensity to an optimal but nonvanishing value [16]. By utilizing this special property of an SR system, it has been widely used in various application areas, such as sequential detectors [20], feed-forward neural network [21], sigma-delta modulators [22], and so on [23].

Mathematically, according to the Linear Response Theory (LRT) of an SR system, when both *d*(*k*) and *η*(*k*) are introduced into the SR system as two independent additive components, and if we set the mean value of the unperturbed state variable ${\langle x\left(k\right)\rangle }_{st}=0$ [9], we have

(18) $${\langle x\left(k\right)\rangle }_{asy}=d_{SR}\left(k\right)+\eta_{SR}\left(k\right),$$

where ${\langle \cdot \rangle }_{st}$ and ${\langle \cdot \rangle }_{asy}$ are the steady state and asymptotic limit of the random process, *d_SR_*(*k*) and *η_SR_*(*k*) is the independent SR system responses to the driving signals *d*(*k*) and *η*(*k*), respectively.

So when the PU signal exists in the sensing channel (under *H*_1_), (18) can also be written as

(19) $${\langle x\left(k\right)\rangle }_{asy}=S_{SR}\left(k\right)+n_{SR}\left(k\right)+\eta_{SR}\left(k\right),$$

where *s_SR_*(*k*) and *n_SR_*(*k*) are the system responses to *s*(*k*) and *n*(*k*), respectively. If the PU signal does not exist (under *H*_0_), it becomes

(20) $${\langle x\left(k\right)\rangle }_{asy}=n_{SR}\left(k\right)+\eta_{SR}\left(k\right).$$

Without loss of generality and to simplify the analyses thereafter, we can set the mean value of ${\langle x\left(k\right)\rangle }_{asy}$ under both hypotheses to be zero, that is

(21) $$\{\begin{array}{cc} H_{0}: & E\left[x\left(k\right)\right]=E\left[n_{SR}\left(k\right)+\eta_{SR}\left(k\right)\right]=0,\\ H_{1}: & E\left[x\left(k\right)\right]=E\left[s_{SR}\left(k\right)+n_{SR}\left(k\right)+\eta_{SR}\left(k\right)\right]=0. \end{array}$$

Simultaneously, the power of both driving signals may be changed according to the Spectrum Power Amplification (SPA) property of the SR system [16], that is

(22) $$\lambda_{s}=\frac{\sigma_{s_{SR}}^{2}}{\sigma_{s}^{2}},$$

(23) $$\lambda_{n}=\frac{\sigma_{n_{SR}}^{2}}{\sigma_{n}^{2}},$$

(24) $$\lambda_{\eta }=\frac{\sigma_{\eta_{SR}}^{2}}{\sigma_{\eta }^{2}},$$

where *λ_s_*, *λ_n_*, *λ_η_* are the SPAs of *s*(*k*), *n*(*k*) and *η*(*k*), respectively; $\sigma_{s_{SR}}^{2}$, $\sigma_{n_{SR}}^{2}$, $\sigma_{\eta_{SR}}^{2}$ are the variances of *s_SR_*(*k*), *n_SR_*(*k*) and *η_SR_*(*k*), respectively.

Then the SNR of the asymptotic limit of *x*(*k*), say *SNR_o_*, can be calculated by

(25) $$SNR_{o}=\frac{\sigma_{s_{SR}}^{2}}{\sigma_{n_{SR}}^{2}+\sigma_{\eta_{SR}}^{2}}.$$

By introducing the above state variable *x*(*k*) of the SR system into the conventional detector as shown in Figure 1, and by normalizing the output signal *y*(*k*) to a variable with unit variance, we have

(26) $$\{\begin{array}{cc} H_{0}: & y\left(k\right)=\frac{{\left[n_{SR}\left(k\right)+\eta_{SR}\left(k\right)\right]}^{2}}{\frac{1}{K}\sum_{k=1}^{K}x^{2}\left(k\right)},\\ H_{1}: & y\left(k\right)=\frac{{\left[s_{SR}\left(k\right)+n_{SR}\left(k\right)+\eta_{SR}\left(k\right)\right]}^{2}}{\frac{1}{K}\sum_{k=1}^{K}x^{2}\left(k\right)}, \end{array}(k=1,2,\cdots ).$$

According to the Central Limit Theorem [3], when the number of samples *K* is large enough, *y*(*k*) under both hypotheses can be approximated by the standard Gaussian distributions with a unit variance but different mean values.

Thus, the conditions in [24] to improve the CR networks spectrum sensing by using the optimal SR can be fulfilled, especially under a low SNR circumstance. Here we also give the corresponding Theorem as follows:

**Theorem** **1.** (also appearing in [24], Theorem 6) *For the weak signal-detection problem, if the test statistics under both hypotheses can be well approximated by some Gaussian distributions with the same variance, then the optimum detection performance can be obtained by adding a constant SR noise to the observed data and adjusting the detector threshold*.

In other words, we can carry out the optimization process as

(27) $$\left\{f^{*}\left[\cdot \right],\eta^{*}\left(k\right),P^{*}\left(k\right)\right\}=\underset{\left\{f\left[\cdot \right],\eta \left(k\right),P\left(k\right)\right\}}{\arg\max}SNR_{o},$$

where $f^{*}\left[\cdot \right]$ is the optimal SR system, $\eta^{*}\left(k\right)$ and $P^{*}\left(k\right)$ are the corresponding optimal SR noise and optimal system parameter vector of $f^{*}\left[\cdot \right]$.

According to Theorem 1, and under the condition of (27), we can get

(28) $$SNR_{o}>SNR_{i},$$

or a SNR gain can be achieved as

(29) $$SNR_{gain}=SNR_{o}-SNR_{i}>0.$$

Calculate the mean value of *y*(*k*) and compare it with a fixed threshold *γ_OSR-ED_* to achieve a CFAR as shown in Figure 1, the spectrum sensing decision can then be made by

(30) $$B(y)=\frac{1}{K}\sum_{k=1}^{K}y\left(k\right)\begin{array}{c} H_{1}\\ >\\ <\\ H_{0} \end{array}\gamma_{OSR-ED}.$$

If there exists a noise uncertainty ρ as in (12), the sampling complexity of the proposed optimal SR-based detection method *N_OSR-ED_* is

(31) $$\begin{array}{l} N_{OSR-ED}=\frac{2{\left[Q^{-1}\left(P_{fa(ED)}\right)-Q^{-1}\left(1-P_{d(ED)}\right)\right]}^{2}}{{\left[SNR_{o}-\left(\rho -\frac{1}{\rho }\right)\right]}^{2}}\\ =\frac{2{\left[Q^{-1}\left(P_{fa(ED)}\right)-Q^{-1}\left(1-P_{d(ED)}\right)\right]}^{2}}{{\left[SNR_{i}-\left(\rho -\frac{1}{\rho }-SNR_{gain}\right)\right]}^{2}} \end{array}$$

Compared with the conventional detector under the same CFAR and detection probability, it can be found obviously based on (13), (29) and (31) that

(32) $$N_{OSR-ED}<N_{ED},$$

which implies that the sampling complexity can be reduced effectively based on the proposed approach. Meanwhile, according to the definition of the SNR wall, it can be defined that the SNR wall of the proposed optimal SR-based detection approach is

(33) $$SNR_{wall}^{\left(OSR-ED\right)}=\rho -\frac{1}{\rho }-SNR_{gain},$$

and we have

(34) $$SNR_{wall}^{\left(OSR-ED\right)}<SNR_{wall}^{\left(ED\right)}.$$

So the SNR wall of the proposed approach can also be improved accordingly.

Simultaneously, if we define the size of the noise uncertainty after the optimal SR-based detection as *ρ*^(*OSR-ED*)^, we have

(35) $$\begin{array}{l} SNR_{wall}^{\left(OSR-ED\right)}=\rho^{\left(OSR-ED\right)}-\frac{1}{\rho^{\left(OSR-ED\right)}}\\ =\rho -\frac{1}{\rho }-SNR_{gain} \end{array},$$

and it can also be deduced from (34) and (35) that

(36) $$\rho^{\left(OSR-ED\right)}<\rho ,$$

which indicates that the noise uncertainty of the received signal after the proposed optimal SR-based detection processing can also be reduced. This can accordingly relieve the SNR wall problem and improve the real application conditions of the conventional energy detection method especially under low SNR.

## 4. Performance Improvement Analyses

In the following Section, we give a more detailed theoretical performance improvement analyses based on the mostly studied overdamped bistable SR system model [16]. Because the performance of the proposed SR-based detection approach and the conventional energy detector had close relationships with the power of the signal, and to simplify the analyses, we first assumed that the PU signal was an M-PSK modulated signal as follows

(37) $$s\left(k\right)=A_{P}\cdot \cos\left(\omega_{P}kT+\varphi_{P}\right),\;(k=1,2,\cdots )$$

where A_P_, ω_P_ and $\varphi_{P}\in \left\{0,\frac{2\pi }{M},\frac{4\pi }{M},\cdots ,\frac{2\left(M-1\right)\pi }{M}\right\}$ are the amplitude, angular frequency and phase of the PU signal, respectively; and T is the sampling time period. The channel noise *n*(*k*) in (1), is assumed to be an additive white Gaussian noise with mean 0.

By introducing the normalized received signal *d*(*k*) and an SR noise *η*(*k*) into the overdamped bistable SR system, the dynamic equation of the SR system can be written as [16]

(38) $$\begin{array}{l} \frac{x\left(k+1\right)-x\left(k\right)}{T}\\ =a\cdot x\left(k\right)-b\cdot x^{3}\left(k\right)+p_{1}\cdot d\left(k\right)+p_{2}\cdot \eta \left(k\right) \end{array}$$

where *a* and *b* are the system parameters, *p*_1_ and *p*_2_ are the driving parameters corresponding to the driving signals *d*(*k*) and *η*(*k*), respectively.

Due to the independency within *x*(*k*), *n*(*k*) and *η*(*k*) under *H*_1_, (38) can be rewritten as

(39) $$\begin{array}{l} \frac{x\left(k+1\right)-x\left(k\right)}{T}=a\cdot x\left(k\right)-b\cdot x^{3}\left(k\right)\\ +p_{1}\cdot \frac{\bar{h}\cdot A_{P}\cdot \cos\left(\omega_{P}kT+\varphi_{P}\right)}{\sqrt{\frac{1}{2}A_{P}^{2}+\sigma_{n}^{2}}}\\ +p_{1}\cdot \frac{n\left(k\right)}{\sqrt{\frac{1}{2}A_{P}^{2}+\sigma_{n}^{2}}}+p_{2}\cdot \eta \left(k\right). \end{array}$$

To reach the optimal SR performance, it is required that the SR noise should be symmetric [24], and in (39) it is obvious that both *η*(*k*) and *n*(*k*) play the role of SR noise simultaneously, so *η*(*k*) can also be chosen as a white Gaussian noise which is independent to *n*(*k*). Under the above assumptions, (39) can be simplified to the following equation

(40) $$\begin{array}{l} \frac{x\left(k+1\right)-x\left(k\right)}{T}\\ =a\cdot x\left(k\right)-b\cdot x^{3}\left(k\right)\\ +p_{3}\cdot A_{P}\cdot \cos\left(\omega_{P}kT+\varphi_{P}\right)+p_{4}\cdot \lambda \left(k\right) \end{array}$$

where we have

(41) $$p_{3}=\frac{p_{1}\cdot \bar{h}}{\sqrt{\frac{1}{2}A_{P}^{2}+\sigma_{n}^{2}}},$$

(42) $$p_{4}=\sqrt{\frac{p_{1}^{2}\cdot \sigma_{n}^{2}}{\frac{1}{2}A_{P}^{2}+\sigma_{n}^{2}}+p_{2}^{2}},$$

and *λ*(*k*) is a standard Gaussian noise with mean 0 and variance 1.

In this case, the SNR of the received signal at the CR networks SU’s receiver is

(43) $$SNR_{i}=\frac{\frac{1}{2}{\bar{h}}^{2}\cdot A_{P}^{2}}{\sigma_{n}^{2}},$$

and the SNR of the output of the SR system, say *SNR_o_* of *x*(*k*), can be calculated by

(44) $$\begin{array}{l} SNR_{o}\\ =\frac{\sqrt{2}ap_{3}^{2}{\bar{h}}^{2}A_{P}^{2}c^{2}}{p_{4}^{4}}e^{-\frac{2U_{0}}{p_{4}^{2}}}\cdot {\left(1-\frac{2p_{3}^{2}{\bar{h}}^{2}A_{P}^{2}c^{2}}{p_{4}^{4}}\right)}^{-1}\\ =\frac{\sqrt{2}ap_{3}^{2}{\bar{h}}^{2}A_{P}^{2}c^{2}}{p_{4}^{4}-2p_{3}^{2}{\bar{h}}^{2}A_{P}^{2}c^{2}}e^{-\frac{2U_{0}}{p_{4}^{2}}} \end{array}$$

where $c=\sqrt{\frac{a}{b}}$ and $U_{0}=\frac{a^{2}}{4b}$ are fixed constants corresponding to the SR system parameters *a* and *b*.

Based on (43) and (44), and to guarantee the SNR improvement through the optimal SR system, it requires *SNR_o_* > *SNR_i_*, that is

(45) $$\frac{\sqrt{2}ap_{3}^{2}{\bar{h}}^{2}A_{P}^{2}c^{2}}{p_{4}^{4}-2p_{3}^{2}{\bar{h}}^{2}A_{P}^{2}c^{2}}e^{-\frac{2U_{0}}{k_{4}^{2}}}>\frac{\frac{1}{2}{\bar{h}}^{2}A_{P}^{2}}{\sigma_{n}^{2}},$$

and it can be deduced that

(46) $$\left(\sqrt{2}U_{0}+\frac{{\bar{h}}^{2}A_{P}^{2}c^{2}}{\sigma_{n}^{2}}e^{\frac{2U_{0}}{p_{4}^{2}}}\right)p_{3}^{2}>\frac{p_{4}^{4}}{2\sigma_{n}^{2}}e^{\frac{2U_{0}}{p_{4}^{2}}}.$$

When *SNR_i_* is low enough, such as less than −10 dB, (46) can be simplified to

(47) $$p_{3}^{2}>\frac{p_{4}^{4}}{2\sqrt{2}U_{0}\sigma_{n}^{2}}e^{\frac{2U_{0}}{p_{4}^{2}}}.$$

It can be observed, obviously that $p_{4}=\sqrt{U_{0}}$ is the maximal point of the right side expression of (47) when *U*_0_ and $\sigma_{n}^{2}$ is fixed, so if the following condition can be fulfilled

(48) $$p_{3}^{2}>\frac{U_{0}}{2\sqrt{2}\sigma_{n}^{2}}e^{2},$$

the SNR improvement, say *SNR_o_* > *SNR_i_*, can be guaranteed effectively.

At the same time, to get the maximum *SNR_o_* of the SR system, we can take (44) as an optimization objective function and suppose that *p*_3_ is a constant, and let

(49) $$\frac{\partial SNR_{o}}{\partial \left(p_{4}^{2}\right)}=0.$$

Then we can find out that the optimal parameter $p_{4}^{2}$ fulfills the following cubic equation

(50) $${\left(p_{4}^{2}\right)}^{3}-U_{0}{\left(p_{4}^{2}\right)}^{2}+2U_{0}p_{3}^{2}{\bar{h}}^{2}A_{P}^{2}c^{2}=0.$$

Or in other words, the optimal parameter *p*_4_ is the solution of the above cubic equation.

By calculating the discriminant Δ of (50), we can get

(51) $$\begin{array}{l} \Delta =U_{0}^{2}p_{3}^{4}{\bar{h}}^{4}A_{P}^{4}c^{4}-\frac{2}{27}U_{0}^{4}p_{3}^{2}{\bar{h}}^{2}A_{P}^{2}c^{2}\\ =U_{0}^{2}p_{3}^{2}{\bar{h}}^{2}A_{P}^{2}c^{2}\left(p_{3}^{2}{\bar{h}}^{2}A_{P}^{2}c^{2}-\frac{2}{27}U_{0}^{2}\right)\\ =U_{0}^{2}p_{3}^{2}{\bar{h}}^{2}A_{P}^{2}c^{2}\left(p_{3}^{2}{\bar{h}}^{2}A_{P}^{2}\frac{a}{b}-\frac{1}{27}\cdot \frac{a^{4}}{8b^{2}}\right)\\ =\frac{U_{0}^{2}ap_{3}^{2}{\bar{h}}^{2}A_{P}^{2}c^{2}}{216b^{2}}\left(216p_{3}^{2}{\bar{h}}^{2}A_{P}^{2}b-a^{3}\right) \end{array}$$

Then the optimization result can be achieved by the power or the amplitude of the driving PU signal, that is:

(a) When $p_{3}\bar{h}A_{P}>\sqrt{\frac{1}{b}{\left(\frac{a}{6}\right)}^{3}}$, Δ>0, the only real solution of (50) is

(52) $$p_{4}^{2}=\frac{U_{0}}{3}+\sqrt[{3}]{\frac{U_{0}^{3}}{27}-U_{0}p_{3}^{2}{\bar{h}}^{2}A_{P}^{2}c^{2}+\sqrt{\Delta }}+\sqrt[{3}]{\frac{U_{0}^{3}}{27}-U_{0}p_{3}^{2}{\bar{h}}^{2}A_{P}^{2}c^{2}-\sqrt{\Delta }};$$

(b) When $p_{3}\bar{h}A_{P}=\sqrt{\frac{1}{b}{\left(\frac{a}{6}\right)}^{3}}$, Δ=0, the real triple solution of (50) is

(53) $$p_{4}^{2}=\frac{U_{0}}{3}+2\sqrt[{3}]{\frac{U_{0}^{3}}{27}-U_{0}p_{3}^{2}{\bar{h}}^{2}A_{P}^{2}c^{2}};$$

(c) When $p_{3}\bar{h}A_{P}<\sqrt{\frac{1}{b}{\left(\frac{a}{6}\right)}^{3}}$, Δ<0, the corresponding three real solutions of (51) are

(54) $$p_{4(1)}^{2}=\frac{U_{0}}{3}+\sqrt[{3}]{\frac{U_{0}^{3}}{27}-U_{0}p_{3}^{2}{\bar{h}}^{2}A_{P}^{2}c^{2}+\sqrt{\Delta }}+\sqrt[{3}]{\frac{U_{0}^{3}}{27}-U_{0}p_{3}^{2}{\bar{h}}^{2}A_{P}^{2}c^{2}-\sqrt{\Delta }},$$

(55) $$\begin{array}{l} p_{4(2)}^{2}=\frac{U_{0}}{3}+\frac{-1+\sqrt{3}i}{2}\cdot \sqrt[{3}]{\frac{U_{0}^{3}}{27}-U_{0}p_{3}^{2}{\bar{h}}^{2}A_{P}^{2}c^{2}+\sqrt{\Delta }}\\ +\frac{-1-\sqrt{3}i}{2}\cdot \sqrt[{3}]{\frac{U_{0}^{3}}{27}-U_{0}p_{3}^{2}{\bar{h}}^{2}A_{P}^{2}c^{2}-\sqrt{\Delta }} \end{array},$$

(56) $$\begin{array}{l} p_{4(3)}^{2}=\frac{U_{0}}{3}+\frac{-1-\sqrt{3}i}{2}\cdot \sqrt[{3}]{\frac{U_{0}^{3}}{27}-U_{0}p_{3}^{2}{\bar{h}}^{2}A_{P}^{2}c^{2}+\sqrt{\Delta }}\\ +\frac{-1+\sqrt{3}i}{2}\cdot \sqrt[{3}]{\frac{U_{0}^{3}}{27}-U_{0}p_{3}^{2}{\bar{h}}^{2}A_{P}^{2}c^{2}-\sqrt{\Delta }} \end{array}.$$

In the two cases (a) and (b) above, *p*_4_ has only one optimal solution; and in the last case (c), we can substitute the three solutions *p*_4(1)_, *p*_4(2)_ and *p*_4(3)_ back into (44), and then find out the optimal one which reaches the maximal value of *SNR_o_*. Because *p*_3_ should satisfy (48), to make the optimization process easier in real applications, we can choose a relatively big value of *p*_3_ to ensure Δ>0, so that the optimization result of *p*_4_ in (52) can be realized.

Substituting the optimal results of *p*_3_ and *p*_4_, which can be expressed by $p_{3}^{*}$ and $p_{4}^{*}$, back into (41) and (42), the optimal driving parameters $p_{1}^{*}$ and $p_{2}^{*}$ in (38) can finally be calculated, which correspond to the driving parameters of the normalized receiving signal *d*(*k*) and the additive SR noise *η*(*k*), respectively.

Simultaneously, by taking $p_{3}^{*}$ and $p_{4}^{*}$ into (44), the optimal output SNR of the SR system can be calculated by

(57) $$SNR_{o}^{*}=\frac{\sqrt{2}ap_{3}^{*2}{\bar{h}}^{2}A_{P}^{2}c^{2}}{p_{4}^{*4}-2p_{3}^{*2}{\bar{h}}^{2}A_{P}^{2}c^{2}}e^{-\frac{2U_{0}}{p_{4}^{*2}}}.$$

According to (33), (43) and (57), the SNR wall of the proposed optimal SR-based detection approach under the size of noise uncertainty *ρ* is

(58) $$\begin{array}{l} SNR_{wall}^{\left(OSR-ED\right)}=\rho -\frac{1}{\rho }\\ -\frac{\sqrt{2}ap_{3}^{*2}{\bar{h}}^{2}A_{P}^{2}c^{2}}{p_{4}^{*4}-2p_{3}^{*2}{\bar{h}}^{2}A_{P}^{2}c^{2}}e^{-\frac{2U_{0}}{p_{4}^{*2}}}+\frac{\frac{1}{2}{\bar{h}}^{2}\cdot A_{P}^{2}}{\sigma_{n}^{2}} \end{array}$$

and the size of the noise uncertainty after the optimal SR-based detection *ρ*^(*OSR-ED*)^ can be calculated by solving the following equation

(59) $$\begin{array}{l} \rho^{\left(OSR-ED\right)}-\frac{1}{\rho^{\left(OSR-ED\right)}}\\ =\rho -\frac{1}{\rho }-\frac{\sqrt{2}ap_{3}^{*2}{\bar{h}}^{2}A_{P}^{2}c^{2}}{p_{4}^{*4}-2p_{3}^{*2}{\bar{h}}^{2}A_{P}^{2}c^{2}}e^{-\frac{2U_{0}}{p_{4}^{*2}}}\\ +\frac{\frac{1}{2}{\bar{h}}^{2}\cdot A_{P}^{2}}{\sigma_{n}^{2}} \end{array}$$

So when the optimal SR is realized, the corresponding sampling complexity of the proposed optimal SR-based detection approach *N_OSR-ED_* under selected *P_fa_*_(*ED*)_ and *P_d_*_(*ED*)_ is

(60) $$\begin{array}{l} N_{OSR-ED}\\ =\frac{2{\left[Q^{-1}\left(P_{fa(ED)}\right)-Q^{-1}\left(1-P_{d(ED)}\right)\right]}^{2}}{{\left[SNR_{i}-\left(\rho^{\left(OSR-ED\right)}-\frac{1}{\rho^{\left(OSR-ED\right)}}\right)\right]}^{2}}\\ =\frac{2{\left[Q^{-1}\left(P_{fa(ED)}\right)-Q^{-1}\left(1-P_{d(ED)}\right)\right]}^{2}}{{\left[SNR_{i}-SNR_{wall}^{\left(OSR-ED\right)}\right]}^{2}} \end{array}$$

Although the above performance improvement analyses and the corresponding derivations in this Section were based on the assumption that the PU signal was an M-PSK signal, it can be discovered very clearly that the total analyses process from Equation (37) to (60) is only related to the signal amplitude *A_P_*, and is not related to the signal angular frequency *ω_P_* and signal phase *φ_P_* of the PU signal at all. So according to this interesting phenomenon, we can get a more general conclusion that the proposed optimal SR-based signal detection approach in CR networks is suitable for any kind of PU signal whose modulation scheme is not related to amplitude modulation. In other words, many classic and typical signals such as the frequency modulation (FM) signal, phase modulation (PM) signal, and orthogonal frequency division multiplexing (OFDM), which is generally used in 4G and 5G mobile communication systems are all suitable for this kind of proposed approach, which also indicates that the proposed approach may have a wide range of application areas.

## 5. Computer Simulations

In this Section, some computer simulations are given to evaluate the performances of the proposed optimal SR-based detection approach and the conventional energy detection method.

In the simulations, a QPSK signal is chosen as the PU signal with *M* = 4 and $\varphi_{P}\in \left\{0,\frac{\pi }{2},\pi ,\frac{3\pi }{2}\right\}$. The amplitude and angular frequency of the QPSK signal are set as *A_P_* = 1 and $\omega_{P}=2\pi \times 10^{6}$ in (37). The signal amplitude *A_P_* can also be set to some other constant value, but it will not influence the performance of the proposed approach, and the reason for selecting *A_P_* = 1 here lies in that it may simplify the signal normalization and the noise variance estimation process at the receiver as shown in the following Equation (61) to (64). The fading factor is chosen as $\bar{h}=1$. The sampling time period is T = 0.0195. In the overdamped bistable SR system, the parameters are chosen as *a* = 2 and *b* = 1 in (38). The parameters chosen for T, a and b here can guarantee the overdamped bistable SR system could possess the best SR performance including SNR improvement and signal power amplification compared with other parameter values [16].

The maximum likelihood estimation (MLE) method in [6] is used to estimate the amplitude *A_P_* of the PU signal at the input angular frequency *ω_P_* especially under low SNR, that is

(61) $${\hat{A}}_{P}=\sqrt{{\hat{\alpha }}_{1}^{2}+{\hat{\alpha }}_{2}^{2}},$$

where we have

(62) $${\hat{\alpha }}_{1}=\frac{2}{K}\sum_{k=1}^{K}r\left(k\right)\cdot \cos\omega_{P}kT,$$

(63) $${\hat{\alpha }}_{2}=\frac{2}{K}\sum_{k=1}^{K}r\left(k\right)\cdot \sin\omega_{P}kT.$$

and the noise variance of the received signal can be estimated by [20]

(64) $${\hat{\sigma }}_{n}^{2}=\frac{1}{K}\sum_{k=1}^{K}r^{2}\left(k\right)-\frac{1}{2}{\hat{A}}_{P}^{2}.$$

By taking the estimates of ${\hat{A}}_{P}$ in (61) and ${\hat{\sigma }}_{n}^{2}$ in (64) to substitute *A_P_* and $\sigma_{n}^{2}$ in the last Section, the optimization process can then be followed.

**Figure 2:**
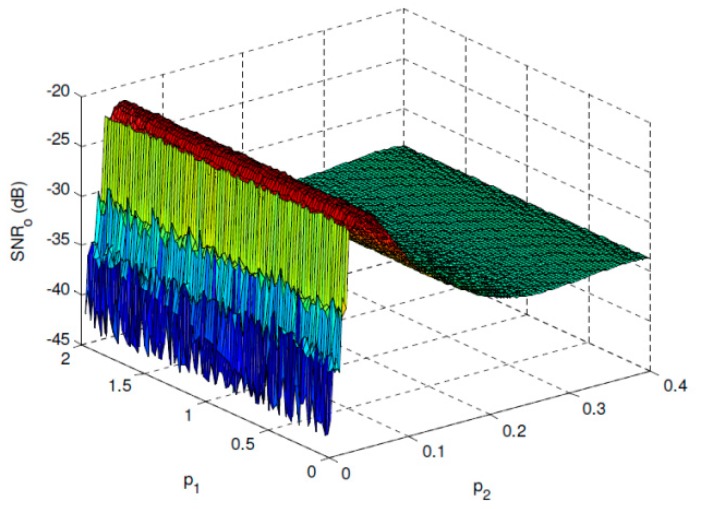
*SNR_o_* performance of the overdamped bistable SR system with *a* = 2 and *b* = 1 under *SNR_i_* = −30 dB.

**Figure 3:**
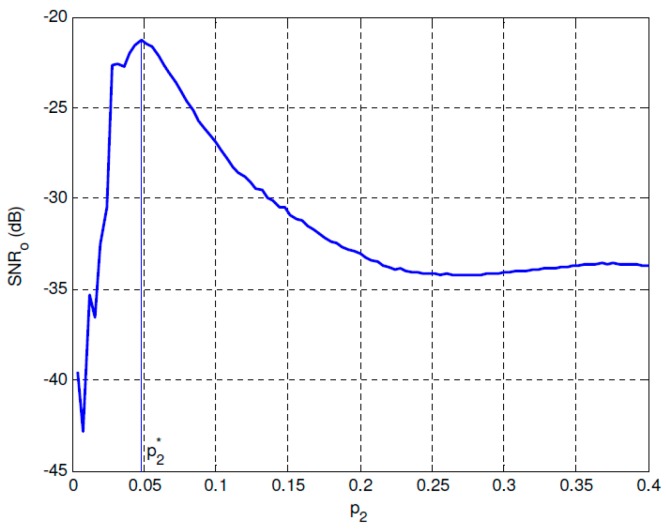
Optimal parameter $p_{2}^{*}$ under *SNR_i_* = −30 dB.

**Figure 4:**
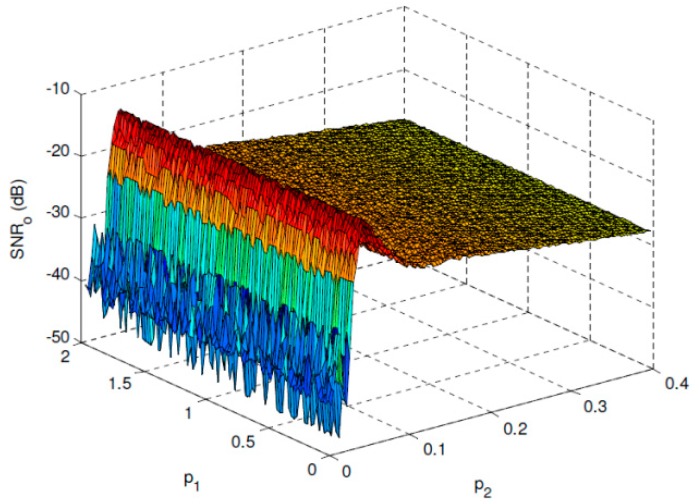
*SNR_o_* performance of the overdamped bistable SR system with *a* = 2 and *b* = 1 under *SNR_i_* = −25 dB.

**Figure 5:**
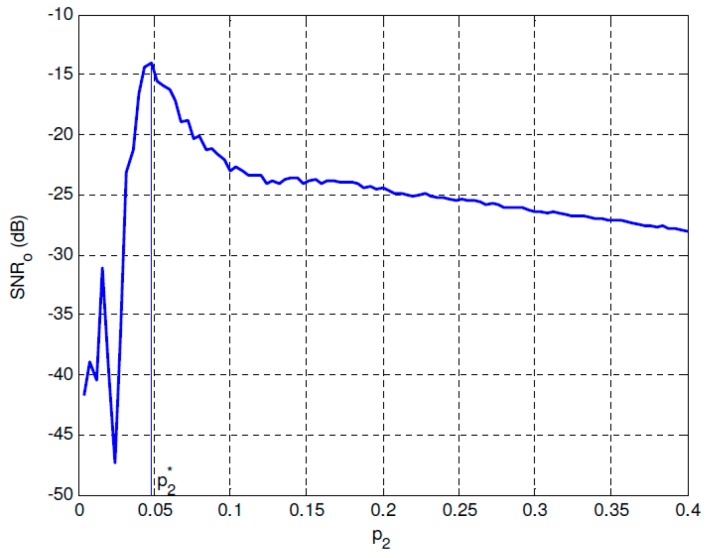
Optimal parameter $p_{2}^{*}$ under *SNR_i_* = −25 dB.

Figure 2 shows the *SNR_o_* performance of the overdamped bistable SR system with parameters *a* = 2 and *b* = 1 under *SNR_i_* = −30 dB. It can be found that a maximal *SNR_o_* can be reached when *p*_2_ approaches the optimal value, and *SNR_o_* has almost no relationship with *p*_1_ when it fulfills the SNR improvement requirement. The optimal performance can be observed much clearly in Figure 3 where *p*_1_ is fixed and $p_{2}^{*}$ is the corresponding optimal value of *p*_2_. Figure 4 and Figure 5 give the same evaluations under *SNR_i_* = −25 dB, which indicates that the proposed optimal SR-based detection approach is efficient even under low SNR, and it can overcome the shortcoming of the conventional energy detection method well.

**Figure 6:**
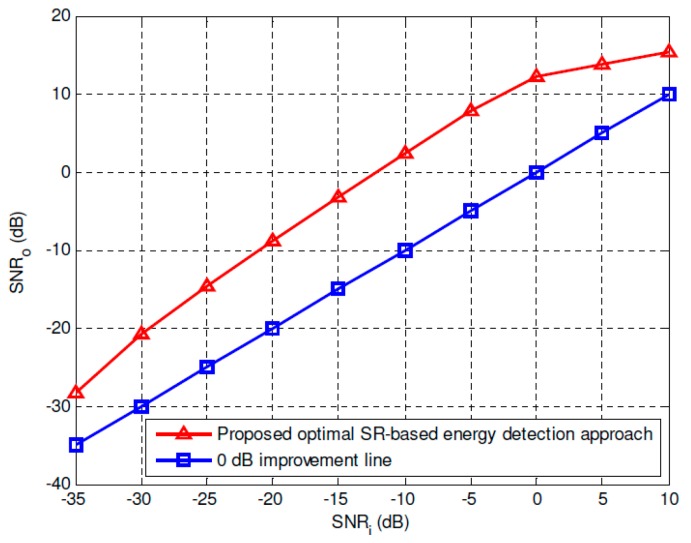
*SNR_o_* vs. *SNR_i_* by using the proposed optimal SR-based detection approach.

**Figure 7:**
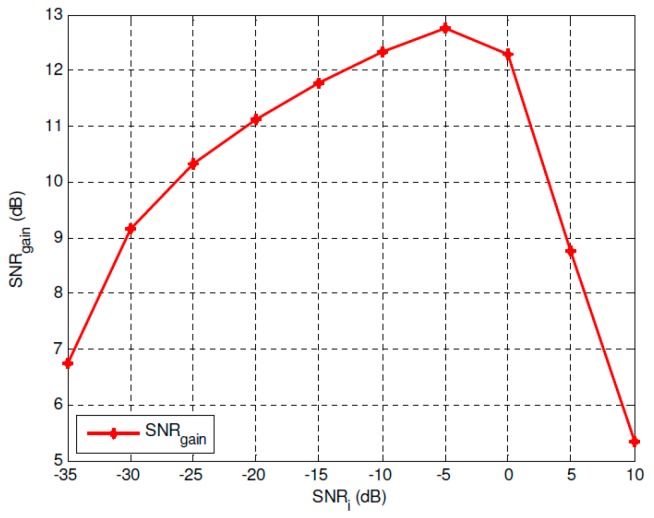
SNR improvement by using the proposed optimal SR approach.

To give a more convincing explanation of the SNR improvement of the proposed optimal SR-based approach, Figure 6 and Figure 7 give the optimal SNR improvement performance of the overdamped bistable SR system with parameters *a* = 2 and *b* = 1. It can be seen that the positive SNR gain can be obtained within a wide range of *SNR_i_* including very low SNR circumstances.

**Figure 8:**
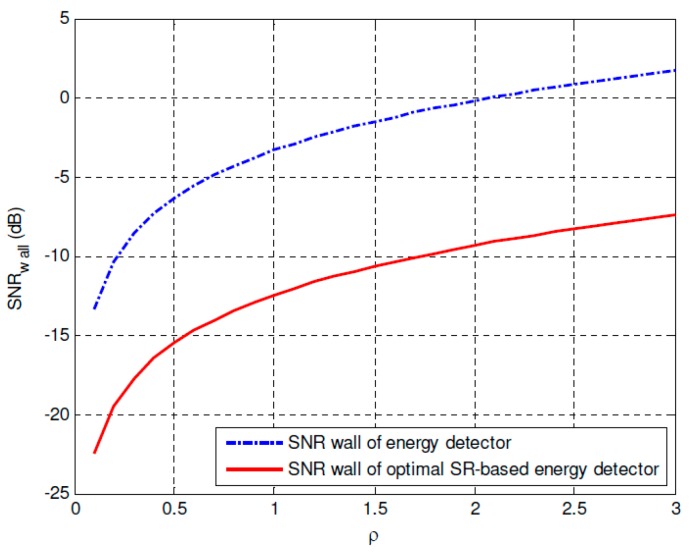
SNR walls comparison under *SNR_i_* = −30 dB.

**Figure 9:**
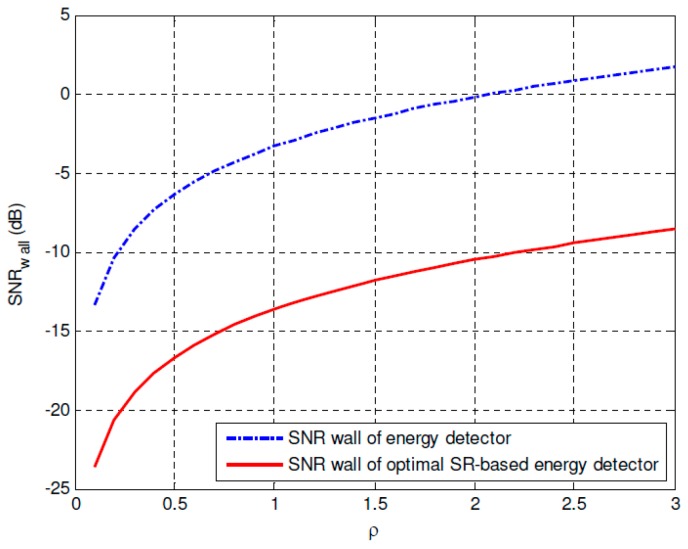
SNR walls comparison under *SNR_i_* = −25 dB.

**Figure 10:**
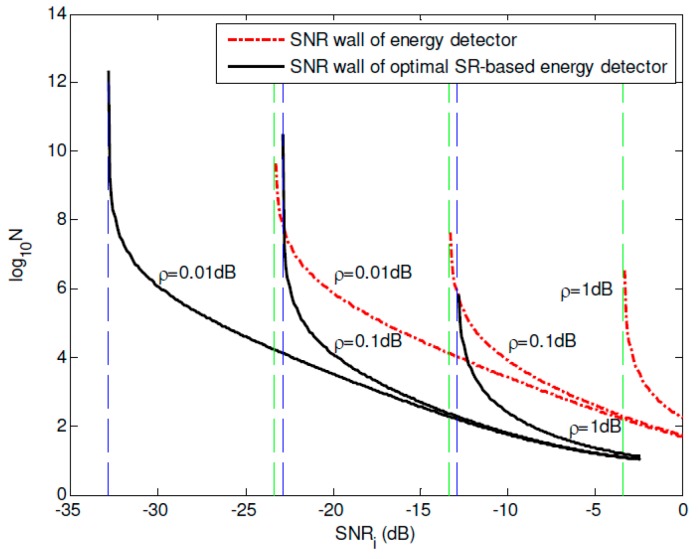
SNR wall comparisons between the energy detector and the optimal SR-based detector under different noise uncertainties.

Figure 8 and Figure 9 compares the SNR walls of the conventional energy detector and the proposed optimal SR-based detector under *SNR_i_* = −30 dB and *SNR_i_* = −25 dB, respectively. It can be found that 9.1424 dB SNR gain under *SNR_i_* = −30 dB and 10.3117 dB SNR gain under *SNR_i_* = −25 dB are obtained by using the proposed approach, which can improve the corresponding spectrum sensing performance of the CR networks importantly. Besides, the SNR wall comparisons between the energy detector and the proposed optimal SR-based detector under different noise uncertainty *ρ* = 1 dB, *ρ* = 0.1 dB and *ρ* = 0.01 dB are plotted in Figure 10. It can be observed that the SNR walls of the proposed approach is much lower than the corresponding SNR walls of the conventional energy detection method under the same noise uncertainty. At the same time, under the same *SNR_i_* and noise uncertainty *ρ*, the sampling complexity can be reduced significantly by using the proposed approach, which can improve the spectrum sensing performance of the energy detector seriously. Or in other words, if we compare the noise uncertainties of both approaches under the same sampling complexity, it can be found that the noise uncertainty of the proposed approach can also be reduced compared with the conventional energy detection method.

**Figure 11:**
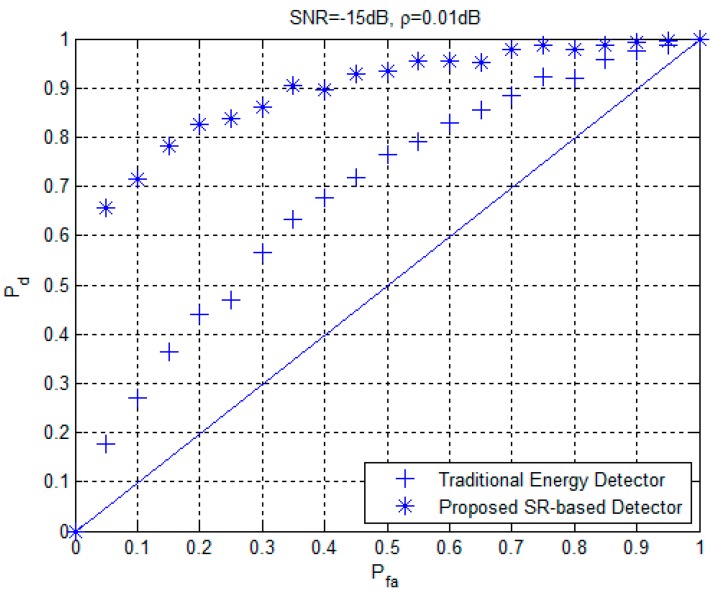
ROC curves of the proposed SR-based detection approach and the traditional energy detector under *SNR* = −15 dB and *ρ* = 0.01 dB.

Finally, to evaluate the validity of the proposed approach based on the SR technique, especially the application in some scenario and implementation in the CR networks, we carried out a computer simulation as follows. A QPSK signal was still selected as the PU signal with *M* = 4 and $\varphi_{P}\in \left\{0,\frac{\pi }{2},\pi ,\frac{3\pi }{2}\right\}$. The SNR was set at −15 dB. A fixed detection threshold was set to maintain the CFAR of PU signal detection. The receiver operating characteristic (ROC) curves of the proposed SR-based detection approach and the traditional energy detector under *SNR* = −15 dB and *ρ* = 0.01 dB is given in Figure 11. As can be found in Figure 11, the detection probabilities of the proposed SR-based detection approach were higher than those of the traditional energy detector, particularly within the range of *P_fa_* value from 0.1 to 0.5, which means that the PU signal can be more easily detected under the fixed threshold by using the proposed approach than the conventional method. In other words, due to the reason that the SNR wall is reduced based on the proposed approach, the PU signal can then be detected with much higher detection probabilities in this condition, while for the conventional energy detection method it still cannot work reliably with much lower detection probabilities. Therefore, it also reveals the real applicability of the proposed approach.

## 6. Conclusions

In this paper, a non-cooperative SR-based detection approach used in CR networks is proposed, and it can relieve the SNR wall and corresponding noise uncertainty problems in traditional energy detectors especially under low SNR circumstances. By introducing the normalized received signal and an independent SR noise into the dynamic SR system as the driving signals, and by optimizing their driving parameters, the SNR of the received signal can be improved and then the SNR wall can be reduced accordingly. Theoretical analyses and computer simulation results verify the effectiveness of the proposed approach. It can enhance the applicability of the traditional energy detectors in real wireless CR networks circumstances efficiently, especially under low SNR circumstances such as deep wireless signal fading, signal shadowing and multipath fading.
